# The utilisation and cost of social care after hip fracture: a prospective observational cohort study

**DOI:** 10.1093/ageing/afaf358

**Published:** 2026-01-24

**Authors:** En Lin Goh, May Ee Png, David Metcalfe, Juul Achten, Duncan Appelbe, Xavier Luke Griffin, Jonathan Alistair Cook, Matthew Lee Costa, WHiTE Investigators, Michael Barrett, Peter Hull, David Melling, Jonathan Kosy, Charalambos P Charalambous, Oliver Keast- Butler, Paul Magill, Rathan Yarlagadda, Girish Vashista, Terence Savaridas, Seb Sturridge, Graham Smith, Kishore Dasari, Deepu Bhaskar, Stefan Bajada, Ewan Bigsby, Ansar Mahmood, Mark Dunbar, Andrea Jimenez, Ryan Wood, James Penny, William Eardley, Robert Handley, Suresh Srinivasan, Matt Gee, Ashwin Kulkarni, John Davison, Mohammad Maqsood, Amit Sharma, Chris Peach, Ahsan Sheeraz, Piers Page, Andrew Kelly, Iain McNamara, Lee Longstaff, Mike Reed, Iain Moppett, Ayman Sorial, Theophilus Joachim, Aaron Ng, Kieran Gallagher, Mark Farrar, Ad Ghande, Jonathan Bird, Shyam Rajagopalan, Andrew McAndrew, Andrew Sloan, Rory Middleton, Ian Dos Remedios, Damian McClelland, Benedict Rogers, James Berstock, Sharad Bhatnagar, Owen Diamond, Paul Fearon, Inder Gill, Doug Dunlop, Tim Chesser, Mehool Acharya, Deepak Sree, Johnathan Craik, David Hutchinson, David Johnson, Mosab Elgalli, Paul Dixon, Pregash Ellapparadja, Guy Slater, Jakub Kozdryk, Jonathan Young, Ben Ollivere, Khitish Mohanty, Mohammad Faisal, Callum Clark, Baljinder Dhinsa, Ibrahim Malek, Sam Heaton, Oliver Blocker, Kanthan Theivendran

**Affiliations:** Oxford Trauma and Emergency Care, Nuffield Department of Orthopaedics, Rheumatology and Musculoskeletal Sciences, University of Oxford, Oxford, UK; Nuffield Department of Primary Care Health Sciences, University of Oxford, Oxford, UK; Oxford Trauma and Emergency Care, Nuffield Department of Orthopaedics, Rheumatology and Musculoskeletal Sciences, University of Oxford, Oxford, UK; Oxford Trauma and Emergency Care, Nuffield Department of Orthopaedics, Rheumatology and Musculoskeletal Sciences, University of Oxford, Oxford, UK; Oxford Trauma and Emergency Care, Nuffield Department of Orthopaedics, Rheumatology and Musculoskeletal Sciences, University of Oxford, Oxford, UK; Bone and Joint Health, Blizard Institute, Queen Mary University of London Barts and The London School of Medicine and Dentistry, London, UK; Oxford Clinical Trials Research Unit, Nuffield Department of Orthopaedics, Rheumatology and Musculoskeletal Sciences, University of Oxford, Oxford, UK; Oxford Trauma and Emergency Care, Nuffield Department of Orthopaedics, Rheumatology and Musculoskeletal Sciences, University of Oxford, Oxford, UK

**Keywords:** hip fracture, social care, complications, cost, older people

## Abstract

**Background:**

The cost of medical care associated with hip fracture has been reported but the cost of social care is less well understood. Social care costs include formal residential and home care and home adaptations, but also informal care from family and friends. This study investigated the utilisation and cost of care beyond acute hospital stays following hip fracture.

**Methods:**

A multi-centre, prospective observational study of patients ≥60 years with a hip fracture in the United Kingdom (UK), with 120-day follow-up. Marginal costs were calculated, with scenario analysis projecting the cost to the UK hip fracture population. A two-part model was used to calculate the incremental mean cost attributable to complications following surgery.

**Results:**

Amongst 16 679 patients with a hip fracture, the mean cost of social care was £15 525 (95% CI: 14 991–16 059) per person. Mean cost per person for the change in residential requirements was £1656 (95% CI: 1568–1743); formal and informal home care £12 849 (95% CI: 12 448–13 250); and home adaptations £1021 (95% CI: 976–1067). The projected national cost of social care in the first 120 days following all hip fractures in the UK was £1.25 billion. Incremental mean cost of social care for patients who developed a surgery-specific or general complication were £1264 (95% CI: 58–2469) and £1418 (95% CI: 792–2043) per person, respectively.

**Conclusion:**

Social care represents a substantial and often under-recognised component of the economic burden following hip fracture. Formal and informal care were major cost drivers after discharge from hospital and may rival or exceed the cost of acute hospital care.

## Key Points

- The utilisation of social care health resources increases dramatically following a hip fracture.
- The projected national cost of social care in the 120 days following all hip fractures in the UK is £1.25 billion.
- Social care represents a substantial and often under-recognised component of the economic burden following hip fracture.

## Introduction

Over 80 000 hip fractures occur every year in the United Kingdom (UK) [1, 2]. The costs associated with the treatment and rehabilitation of these injuries are substantial [3]. Previous reports have indicated that the main cost driver is hospital stay [4], although a significant proportion of resource utilisation can be attributed to the development of post-operative complications [5]. This is unsurprising given that most patients with a hip fracture undergo surgery and patients with frailty are at increased risk of developing complications [6–9]. However, these reports are likely to have underestimated the cost of care after discharge from hospital [10]. This is because the costs associated with social care, especially that of formal and informal care are difficult to measure and therefore, not routinely considered in health economic analyses [5].

There is profound loss of function and independence after a hip fracture [11, 12], with most people requiring additional support after discharge from the hospital [13]. The level of support required varies for each person and is influenced by their overall health and immediate home environment. This typically involves a combination of formal (paid) or informal (unpaid) care and home adaptations. These are significant cost drivers but have been poorly quantified when estimating the overall cost associated with hip fracture care [5]. To date, no health economic evaluation of any hip fracture population has considered formal and informal care as part of their analyses. Furthermore, there is little to no data on the social care costs associated with complications after hip fracture [4, 14].

The aim of this study was to investigate the utilisation and cost of social care incurred by (i) patients with a hip fracture; and (ii) amongst patients who developed complications after a hip fracture.

## Methods

### Study design

The World Hip Trauma Evaluation (WHiTE) study was a multi-centre, prospective observational cohort study that collected data on the assessment, treatment and recovery of a comprehensive hip fracture cohort across 77 participating National Health Service (NHS) hospitals in the England, Wales, and Northern Ireland [15]. Patients were followed up for 120 days after surgery with telephone interviews and postal questionnaires. Recruitment for the study commenced on 8 May 2014, and finished on 29 July 2021, with follow-up to 26 November 2021. Health resource use data collection commenced on 30 March 2017, so only patients who were followed up from this date were included in this study.

### Participants

Patients were eligible for inclusion if they were 60 years or older and received operative treatment for their hip fracture. On enrolment, they received treatment under a standardised care pathway based on the National Institute for Health and Care Excellence Hip Fracture Guidelines [CG124] [16].

### Ethics approval

Ethics approval was granted by the London–Camberwell St Giles Research Ethics Committee. This study was registered with the National Institute of Health Research Portfolio (UKCRN ID12351) and the ISRCTN registry (ISRCTN63982700). Written consent to participate in the study was obtained from all patients. Those who lacked capacity to consent to participate were still included following consultation with their carers.

### Data source

The WHiTE dataset contains data on a core outcome set of patient-reported outcome measures in addition to variables that are routinely measured by the UK National Hip Fracture Database (NHFD) [1]. The full list of variables and outcomes collected as part of the study has been described previously [15]. Data were stored on the OpenClinica V3·7 data collection system (OpenClinica LLC, Waltham, MA, USA).

### Complications

Data on surgery-specific and general complications within 120 days of surgery were collected. The complications of interest were prespecified and are described in Appendix S1 in the Supplementary Data [6, 15]. Complications were recorded from entries made by the treating team in the patient medical records. Patient- or carer-reported complications after discharge from hospital were cross-referenced with medical records from hospital and community databases.

### Health resource use data

Data on resource use at baseline (to represent pre-injury status) and during the first 120 days after hip fracture were collected, which included residential status; formal full- or part-time home care; informal care if the patient was not in residential or nursing home or hospital; and home adaptations. At baseline, patients were asked if they had been living in their own home, a residential or nursing home or hospital in the 120 days prior; and whether they received formal full- or part-time home care or informal care if they were living in their own home in the 120 days prior. At the 120-day follow-up, patients were asked the same questions, and if they had received home adaptations.

### Cost of health resources

The unit costs of health resources were obtained from the Personal Social Services Research Unit Costs of Health [17, 18]. These are presented in Appendix S2 in the Supplementary Data. Unit costs were adjusted to 2022/2023 prices using the 2023 NHS Hospital and Community Health Services (HCHS) Index for health services resources as necessary [18]. The estimated cost of each health resource item was calculated by multiplying the frequency of resource use by the unit cost of that resource and expressed in 2022/2023 British Pound Sterling (£). The assumptions made during the cost estimation were based on previous work by Png *et al.* [10].

### Statistical analysis

The marginal costs, i.e. the costs attributable to hip fracture, were calculated by measuring the difference in utilisation of health resources pre- and post-injury. Descriptive statistics such as means with standard deviations (SD) or 95% confidence intervals (CI) for continuous measures, and proportions (binary) are presented. Available case analysis was performed; this refers to the analysis of data from patients who completed the baseline and/or 120-day health resource section. Statistical analyses were performed with R statistical software (v4.5.0; R Core Team 2025) [19].

####  Analysis of the utilisation and cost of social care associated with hip fracture

Student’s *t*-test was used to calculate the means and 95% CI for each health resource category. The costs of formal and informal care were calculated by multiplying the number of patients who received formal and informal care as a result of their hip fracture by the total number of hours of care received. Sensitivity analysis using complete cases was conducted for comparison of the estimated costs; this refers to the analysis of data from patients who completed both the baseline and 120-day health resource section. A scenario analysis involving the computation of projected social care costs over 120 days for the UK hip fracture population was performed by multiplying the cumulative incidence of hip fracture in 2022 (80 540 patients as reported by the NHFD and Scottish Hip Fracture Audit) by the cost per patient [1, 2].

####  Analysis of the incremental costs of social care associated with complications after hip fracture

The average marginal estimate, which is the incremental cost attributable to a complication after hip fracture, was calculated using the two-part model (TPM), after adjusting for co-variates that may influence heath resource utilisation and therefore, costs. The co-variates selected for inclusion in the statistical models were specified *a priori* and described in detail in Appendix S3 in the Supplementary Data. In the first part of the model, the probability of incurring a cost due to a complication during the 120-day follow-up period was estimated using a logistic regression model. In the second part of the model, the estimated cost conditional on having developed a complication was estimated using a generalised linear model that has a gamma distribution with a log link [20]. TPMs were fitted using the ‘twopartm’ R package [20, 21].

## Results

### Population

There were 24 523 patients enrolled in the WHiTE study, of which 16 679 (68%) patients provided health economic data, with complete follow-up in 14 070 (84%) patients. The mean age of the overall cohort was 82.9 (SD 8.4) years and 11 543 (69%) patients were female. The baseline demographics of the cohort are reported in Table 1.

**Table 1:** Baseline demographics of the WHiTE cohort.

Characteristic		Overall (*n* = 16 679)
Age (SD)		82.9 (8.4)
Gender (%)	Male	5136 (30.8)
	Female	11 543 (69.2)
Regular smoker (%)	Yes	1500 (9.0)
	No	15 092 (90.5)
	Missing	87 (0.5)
Weekly alcohol consumption (%)	0 to 7 units	14 702 (88.1)
	8 to 14 units	1071 (6.4)
	15 to 21 units	356 (2.1)
	>21 units	416 (2.5)
	Missing	134 (0.8)
Diabetic (%)	Yes	2609 (15.6)
	No	14 034 (84.1)
	Missing	36 (0.2)
Renal failure (%)	Yes	1383 (8.3)
	No	15 238 (91.4)
	Missing	58 (0.3)
Cognitive impairment (%)	Yes	5388 (32.3)
	No	10 550 (63.3)
	Missing	741 (4.4)
Residential status (%)	Own home	13 971 (83.8)
	Residential or nursing care	2463 (14.8)
	Rehabilitation unit	86 (0.5)
	Acute hospital	85 (0.5)
	Other	69 (0.4)
	Missing	0 (0.0)
ASA classification (%)	Grade I	285 (1.7)
	Grade II	3499 (21.0)
	Grade III	9467 (56.8)
	Grade IV	2586 (15.5)
	Grade V	55 (0.3)
	Missing	787 (4.7)
Fracture type (%)	Femoral neck—undisplaced (B1)	934 (5.6)
	Femoral neck—displaced (B3)	10 977 (65.8)
	Trochanteric—simple (A1)	1501 (9.0)
	Trochanteric—unstable (A2)	2155 (12.9)
	Trochanteric—transtrochanteric (A3)	542 (3.2)
	Subtrochanteric	544 (3.3)
	Missing	26 (0.2)

### Utilisation rates of social care resources

The utilisation rates of social care resources in the 120 days after hip fracture are presented in Table 2. After the index treatment for their hip fracture, 1602 (7%) patients had a step-up in residential requirements. Amongst those who returned to their own home, 9481 (93%) patients had increased formal and informal care use; and 6194 (61%) patients had a least one form of home adaptation. Results of the sensitivity analysis using complete cases are presented in Appendix S4 in the Supplementary Data.

**Table 2:** Utilisation rate of social care resources and estimated cost per patient over 120 days of follow-up (in 2022/23 £) using available case analysis.

Resource category	Item	Number of patients (%)	Number of events/hours/units	Mean cost per patient (£)	95% CI	Total cost (£)	Mean cost (£)	95% CI
Residential status	Residential home	599 (4.3)	17	705.67	650.26–761.09	23 293 519	1656	1568–1743
	Nursing home	824 (5.9)	17	949.87	879.20–1020.54			
Formal and informal home care	Full-time home care	183 (1.3)	522 648	1336.10	1144.24–1527.96	130 426 200	12 849	12,448–13,250
	Part-time home care	4025 (29.9)	389 107	1034.96	977.32–1092.60			
	Informal home care	5273 (37.5)	6 111 489	10 477.55	10 112.95–10 842.15			
Home adaptations	Bedroom	260 (1.8)	260	£25.24	22.22–28.27	10 365 969	1021	976–1067
	Bathroom	1010 (7.2)	1010	£540.33	508.70–571.95			
	Level access shower	260 (1.8)	260	£161.35	141.99–180.72			
	Toilet	178 (1.3)	178	£52.90	45.20–60.61			
	Stairlift	596 (4.2)	596	£149.03	137.42–160.64			
	Fixed hoist	79 (0.6)	79	£29.58	23.08–36.08			
	Grab rails	2852 (20.3)	2852	£32.87	31.85–33.90			
	Outdoor rails	675 (4.8)	675	£9.35	8.67–10.03			
	Ramp	182 (1.3)	182	£12.87	11.02–14.72			
	Steps	102 (0.7)	102	£7.63	6.16–9.11			

### Cost of social care resource utilisation

The costs of social care resource utilisation in the 120 days after hip fracture are presented in Table 2. The total cost of social care for hip fracture in the first 120 days was £164 085 688 [mean cost of £15 525 per person (95% CI: 14 991–16 059)]. The cost relating to the utilisation of social care resources in the 120 days after hip fracture associated with the change in residential requirements was £23 293 519 [mean cost of £1656 per person (95% CI: 1568–1743)]; formal and informal home care £130 428 940 [mean cost of £12 849 per person (95% CI: 12 448–13 250)]; and home adaptations £10 365 969 [mean cost of £1021 per person (95% CI: 976–1067)] in total, on average.

In the sensitivity analysis, the total cost of social care for hip fracture in the first 120 days after hip fracture was £150 114 375 [mean cost of £15 748 per person (95% CI: 15 184–16 313)]. The mean cost per person associated with the change in residential requirements, formal and informal home care, and home adaptations were similar in both analyses. In the scenario analysis, the projected cost of social care of all hip fractures in the UK during the first 120 days was estimated at £1.25 billion (95% CI: 1.21 billion–1.29 billion).

### Incremental costs of social care associated with complications

**Figure 1:**
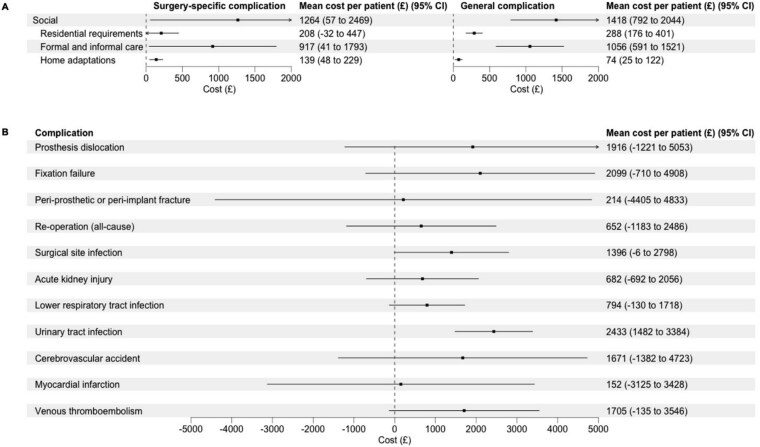
Forest plots showing the adjusted incremental mean cost associated with complications after hip fracture for each social care resource category and the adjusted incremental mean cost of social care associated with individual complications. (A) mean cost per patient for a surgery-specific or general complication across social care resource categories; (B) mean cost per patient for social care for individual complications.

The incremental mean costs of a surgery-specific or general complication on each resource category of social care are reported in Table 3 and Figure 1. Patients who developed a surgery-specific complication incurred higher mean cost of social care of £1264 (95% CI: 58–2469) compared with those who did not. The incremental mean cost associated with the change in residential requirements was £208 (95% CI: −32–447); formal or informal home care £917 (95% CI: 41–1793); and home adaptations £139 (95% CI: 48–229). Patients who developed a general complication incurred higher mean cost of social care of £1418 (95% CI: 792–2043) compared with those who did not. The incremental mean cost associated with the change in residential requirements was £288 (95% CI: 176–401); formal or informal home care £1056 (95% CI: 591–1521); and home adaptations £74 (95% CI: 25–122).

**Table 3:** Adjusted incremental mean cost associated with complications after hip fracture for each social care resource category.

Resource category	Surgery-specific complication			General complication		
	Mean cost per patient (£)	95% CI	*P-*value	Mean cost per patient (£)	95% CI	*P*-value
Residential status	208	−32–447	.089	288	176–401	<.001
Formal and informal home care	917	41–1793	.040	1056	591–1521	<.001
Home adaptations	139	48–229	.003	74	25–122	.003

The incremental mean costs of social care associated with specific complications are reported in Figure 1. The incremental mean cost associated with prosthesis dislocation was £1916 (95% CI: −1221–5053); fixation failure £2099 (95% CI: −710–4908); peri-prosthetic or peri-implant fracture £214 (95% CI: −4405–4833); re-operation (all-cause) £625 (95% CI: −1183–2486); surgical site infection £1396 (95% CI: −6–2798); acute kidney injury £682 (95% CI: −692–2056); lower respiratory tract infection £794 (95% CI: −130–1718); urinary tract infection £2433 (95% CI: 1482–3384); cerebrovascular accident £1671 (95% CI: −1382–4723); myocardial infarction £152 (95% CI: −3125–3428); and venous thromboembolism £1705 (95% CI: −135–3546).

## Discussion

This cost-of-illness study estimated the social care cost amongst patients with hip fracture at £164 million. Projecting this to the UK hip fracture population, the total cost of social care of all hip fractures during the first 120 days is estimated at £1·25 billion.

The utilisation of social care resources increased in the 120 days after hip fracture. Approximately 3178 (22%) patients were in a residential or nursing home, rehabilitation unit or hospital 120 days after their injury, representing a 21% increase from before their injury. As a result of their hip fracture, 4208 (41%) and 5273 (52%) patients reported new formal and informal care needs, respectively. Of the patients who received formal care, 2662 (63%) were funded via the NHS, 930 (22%) were funded privately and 369 (9%) were funded by a combination of NHS and privately. Informal care utilisation, from family and friends, was high and the utilisation and cost of this significantly outweighed that of formal care. More than half of the patients living in their own home reported having an informal carer after their hip fracture. This utilisation rate is higher than previously reported by Png *et al.* [10], which is likely due to the larger population size of the present cohort. While formal and informal care can substitute for each other [22–24], spending on formal care may reduce the need for more expensive informal care, resulting in cost savings [25].

Leal *et al.* estimated that the mean cost of hospital treatment during the first year after hip fracture was £8613 (in 2012/2013 prices) [4]; or £10 290 (in 2022/2023 prices) after adjusting for inflation using the 2023 HCHS index [18]. If we assume the same consumption rate for care homes and formal and informal home care between four months and one year, with the utilisation of medical care (relating to the hip fracture) based on the consumption pattern observed by Leal *et al.* [4], the projected mean health and social care cost would be £56 865 per person. Williamson *et al.* estimated the health and social care cost in the first year after a hip fracture at $43 669 (or £37 153 based on an average exchange rate of $1.00:£0.82 in 2023) [26] per person in their systematic review [5]. The studies included in this review did not consider the cost arising from informal care, which is a likely explanation for the difference observed with our analysis.

Our findings provide further insight into the impact of complications on the costs of social care, which has not been studied previously. Knauf *et al.* reported an incremental cost of medical care of €1566 and €4968 (or £1593 and £5054 based on an average exchange rate of €1.00:£0.89 in 2023) with medical and surgical complications [14], respectively. However, the costs attributable to individual complications were not reported in their analysis. Our data indicate that the cost of social care is similar to the cost of medical care for general complications but lower than the cost of medical care for surgery-specific complications. It is evident that the costs associated with complications continue to accrue even after the index hospital admission, for which the utilisation of formal and informal care are significant cost drivers. These are ‘hidden costs’ that are not recorded in national datasets and not routinely considered in health economic analyses. When these costs are accounted for, the true burden of health and social care expenditure following hip fracture is substantially higher, as reported in this study.

A key strength of this study is the data on formal and informal care and home adaptations, which have not been measured in previous observational studies of hip fracture populations [4, 27, 28]. The WHiTE cohort has been shown to be representative of the wider population of hip fractures in the UK and comparable to other populations worldwide [6, 29]. Furthermore, our exploration of the relationship between complications and social care costs provide insight into the burden of complications following hip fracture. We have previously shown that complications are influenced by treatment-related factors such as the type of operation, delayed surgery, and delayed mobilisation after surgery, and may therefore be preventable [8, 9]. As such, it is possible that a proportion of the utilisation and cost of social care attributable to complications after hip fracture is potentially avoidable. Consideration should also be given that complications may be due to unmet social care needs arising from the inadequate provision of support. However, there are important limitations that should be considered. We did not explore the association between socioeconomic status and social care utilisation as we did not collect socioeconomic data. It is plausible that socioeconomic status may shape the need for and access to social care, which would be an area of exploration in future work. The diagnoses of complications made by treating clinicians were accepted in the knowledge that these may be subject to surveillance bias, which can result in over- or under-reporting. The questionnaire design meant that several assumptions were made that could under- or overestimate the cost of health resource utilisation [10]. Patients who died before the end of follow-up were treated as a non-response instead of assuming they had not incurred any cost; those who reported living in their own home were assumed to have been there since discharge and vice versa for those who reported not living in their own home. Since social care needs and provision are not always accurately captured, costs of medical and formal care may be either under- or overestimated. For example, some patients with unmet needs may lead to an underestimation of true resource requirements, while imputation methods or assumptions about service use may result in overestimation [10]. This challenge is not unique to our study and should be considered when interpreting any health economic analysis [30].

## Conclusion

Social care represents a substantial and often under-recognised component of the economic burden following hip fracture. The utilisation of social care resources and associated costs were high, amounting to £1.25 billion in the first 120-days when projected to the UK hip fracture population. Formal and informal care were the major cost drivers after discharge from hospital and may rival or exceed the cost of acute hospital care. These findings underscore the need for integrated health and social care planning to support recovery in older adults.

## Supplementary Material

Supplementary_materials_afaf358

## Data Availability

All data requests should be submitted to the corresponding author for consideration.
