# Supplementary material for: The utilisation and cost of social care after hip fracture: a prospective observational cohort study
Source: Age Ageing. 2026 Jan 24;55(1):afaf358. doi: 10.1093/ageing/afaf358 (PMC12831183; doi:10.1093/ageing/afaf358)
Supplement: Supplementary_materials_afaf358 [file supplementary_materials_afaf358.docx]

**The utilisation and cost of social care after hip fracture: a prospective observational cohort study**

**Supplementary Data**

[**Appendix 1. Complications 2**](#_Toc213409938)

[**Appendix 2. Reference costs 3**](#_Toc213409939)

[**Appendix 3. Co-variates 4**](#_Toc213409940)

[**Appendix 4. Sensitivity analysis 5**](#_Toc213409941)

[**Appendix 5. STROBE statement 6**](#_Toc213409942)

# **Appendix 1. Complications**

**Supplementary Table 1. List of pre-specified complications**

| Classification | Complication |
| --- | --- |
| Surgery-specific | Prosthesis dislocation, fixation failure, peri-prosthetic or peri-implant fracture, re-operation (all-cause), and surgical site infection |
| General | Acute kidney injury, lower respiratory tract infection, urinary tract infection, cerebrovascular accident, myocardial infarction, and venous thromboembolism |

# **Appendix 2. Reference costs**

**Supplementary Table 2. Unit cost of health resources (2022/2023 £)**

| Resource category | Description | Unit | Unit cost (£) | Source |
| --- | --- | --- | --- | --- |
| Residential | Residential home | Per week | 983·00 | PSSRU 2023 p.8[1] |
|  | Nursing home | Per week | 1329·00 | PSSRU 2023 p.7[1] |
| Formal home care | Full-time home care | Per hour | 25·95 | UKHCA, 2023[2] |
|  | Part-time home care | Per hour | 27·00 | PSSRU 2023 p.78[1] |
| Informal home care | Median wage (unknown) | Per hour | 17·93 | Statistica, 2023[3] |
|  | Median wage (male) | Per hour | 16·65 | Statistica, 2023[3] |
|  | Median wage (female) | Per hour | 18·14 | Statistica, 2023[3] |
| Home adaptation† | Bedroom | Per item | 985·62 | PSSRU 2020 p.90[4] |
|  | Bathroom | Per item | 5430·56 | Garrett, 2016: Table 1[5] |
|  | Level-access shower | Per item | 6299·67 | PSSRU 2020 p.89[4] |
|  | Toilet | Per item | 3016·98 | Garrett, 2016: Table 1[5] |
|  | Stairlift | Per item | 2538·32 | PSSRU 2020 p.89[4] |
|  | Fixed hoist | Per item | 3801·39 | Garrett, 2016: Table 1[5] |
|  | Grab rail | Per item | 117·01 | PSSRU 2020 p.90[4] |
|  | Outdoor rail | Per item | 140·64 | PSSRU 2020 p.90[4] |
|  | Ramp | Per item | 717·84 | PSSRU 2020 p.90[4] |
|  | Steps | Per item | 759·47 | PSSRU 2020 p.90[4] |

†Inflated to 2022/23 cost using the 2023 NHS Hospital and Community Health Services (HCHS)[1]

PSSRU, Personal Social Services Research Unit, UKHCA, United Kingdom Homecare Association

1. Jones KC, Weatherly H, Birch S *et al.* Unit Costs of Health and Social Care 2023. 2023.

2. Homecare Association. A Minimum Price for Homecare 2022-2023. 2023.

3. Clark D. Median hourly earnings for full-time employees in the United Kingdom from 1997 to 2023, by gender. 2024 https://www.statista.com/statistics/280626/median-hourly-earnings-for-full-time-employees-in-the-uk-by-gender/ (7 September 2024, date last accessed).

4. Curtis LA, Burns A. Unit Costs of Health and Social Care 2020. 2020.

5. Garrett H, Roys M, Burris S, Nicol S. The cost-benefit to the NHS arising from preventative housing interventions. IHS BRE Press Bracknell, 2016.

# **Appendix 3. Co-variates**

**Supplementary Table 3. List of co-variates included in the regression models**

| Variable | Options | Model specification |
| --- | --- | --- |
| Age | Linear | Continuous |
| Sex | Male / Female | Binary |
| Regular smoker | Yes / No | Binary |
| Diagnosis of diabetes | Yes / No | Binary |
| Diagnosis of renal failure | Yes / No | Binary |
| Diagnosis of cognitive impairment | Yes / No | Binary |
| American Society of Anesthesiologists (ASA) classification | ASA grade ≥3 / ASA grade <3 | Binary |
| Fracture type | Femoral neck (undisplaced) / Femoral neck (displaced) / Trochanteric (stable) / Trochanteric (unstable) / Subtrochanteric | Categorical (5 indicator variables) |
| Operation type | Sliding hip screw / Cephalomedullary nail / Cannulated screws / Hip hemiarthroplasty / Total hip arthroplasty | Categorical (4 indicator variables) |

# **Appendix 4. Sensitivity analysis**

**Supplementary Table 4. Utilisation rate of social care health resources and estimated cost per patient over four-months follow-up (in 2022/23 £) using complete case analysis**

| Resource category | Item | Number of patients (%) | Number of events/hours/units | Mean cost per patient (£) | 95% CI | Total cost (£) | Mean cost (£) | 95% CI |
| --- | --- | --- | --- | --- | --- | --- | --- | --- |
| Residential status | Residential home | 585 (4·9) | 17 | 805·55 | 741·74–869·37 | 22,844,464 | 1,898 | 1,797–1,999 |
|  | Nursing home | 813 (6·8) | 17 | 1,102·72 | 1,021·12–1,184·32 |  |  |  |
| Formal and informal home care | Full-time home care | 139 (1·5) | 396,984 | 1,121·09 | 936·10–1,306·09 | 117,794,002 | 12,819 | 12,402–13,236 |
|  | Part-time home care | 3,662 (39·9) | 359,896 | 1,057·48 | 999·83–1,115·14 |  |  |  |
|  | Informal home care | 4,883 (53·1) | 5,618,781 | 10,640·42 | 10,267·73–11,013·11 |  |  |  |
| Home adaptations | Bedroom | 240 (2·6) | 240 | 25·74 | 22·53–28·96 | 9,476,182 | 1,031 | 984–1,079 |
|  | Bathroom | 920 (10·0) | 920 | 543·71 | 510·37–577·04 |  |  |  |
|  | Level access shower | 245 (2·7) | 245 | 167·96 | 147·21–188·72 |  |  |  |
|  | Toilet | 159 (1·7) | 159 | 52·20 | 44·16–60·25 |  |  |  |
|  | Stairlift | 542 (5·9) | 542 | 149·72 | 137·49–161·95 |  |  |  |
|  | Fixed hoist | 65 (0·7) | 65 | 26·89 | 20·37–33·40 |  |  |  |
|  | Grab rails | 2,699 (29·4) | 2,699 | 34·37 | 33·28–35·46 |  |  |  |
|  | Outdoor rails | 627 (6·8) | 627 | 9·60 | 8·87–10·32 |  |  |  |
|  | Ramp | 167 (1·8) | 167 | 13·05 | 11·09–15·01 |  |  |  |
|  | Steps | 97 (1·1) | 97 | 8·02 | 6·43–9·60 |  |  |  |

# **Appendix 5. STROBE statement**

**Supplementary Table 5. STROBE statement**

|  | Item No. | Recommendation | Page  No. |
| --- | --- | --- | --- |
| **Title and abstract** | 1 | (*a*) Indicate the study’s design with a commonly used term in the title or the abstract | 1 |
|  |  | (*b*) Provide in the abstract an informative and balanced summary of what was done and what was found | 3-4 |
| Introduction | | | |
| Background/rationale | 2 | Explain the scientific background and rationale for the investigation being reported | 6 |
| Objectives | 3 | State specific objectives, including any prespecified hypotheses | 6 |
| **Methods** |  |  |  |
| Study design | 4 | Present key elements of study design early in the paper | 7 |
| Setting | 5 | Describe the setting, locations, and relevant dates, including periods of recruitment, exposure, follow-up, and data collection | 7 |
| Participants | 6 | (*a*) Give the eligibility criteria, and the sources and methods of selection of participants. Describe methods of follow-up | 7 |
|  |  | (*b*) For matched studies, give matching criteria and number of exposed and unexposed | – |
| Variables | 7 | Clearly define all outcomes, exposures, predictors, potential confounders, and effect modifiers. Give diagnostic criteria, if applicable | 8-9 |
| Data sources/ measurement | 8 | For each variable of interest, give sources of data and details of methods of assessment (measurement). Describe comparability of assessment methods if there is more than one group | 8-9 |
| Bias | 9 | Describe any efforts to address potential sources of bias | 9-10 |
| Study size | 10 | Explain how the study size was arrived at | – |
| Quantitative variables | 11 | Explain how quantitative variables were handled in the analyses. If applicable, describe which groupings were chosen and why | 9-10 |
| Statistical methods | 12 | (*a*) Describe all statistical methods, including those used to control for confounding | 9-10 |
|  |  | (*b*) Describe any methods used to examine subgroups and interactions | – |
|  |  | (*c*) Explain how missing data were addressed | 9-10 |
|  |  | (*d*) If applicable, explain how loss to follow-up was addressed | – |
|  |  | (*e*) Describe any sensitivity analyses | 9-10 |
| **Results** |  |  |  |
| Participants | 13 | (a) Report numbers of individuals at each stage of study—eg numbers potentially eligible, examined for eligibility, confirmed eligible, included in the study, completing follow-up, and analysed | 10 |
|  |  | (b) Give reasons for non-participation at each stage | 10 |
|  |  | (c) Consider use of a flow diagram | – |
| Descriptive data | 14 | (a) Give characteristics of study participants (eg demographic, clinical, social) and information on exposures and potential confounders | 22 |
|  |  | (b) Indicate number of participants with missing data for each variable of interest | 22 |
|  |  | (c) Summarise follow-up time (eg, average and total amount) | – |
| Outcome data | 15 | Report numbers of outcome events or summary measures over time | 11-12, 23-24 |
| Main results | 16 | (*a*) Give unadjusted estimates and, if applicable, confounder-adjusted estimates and their precision (eg, 95% confidence interval). Make clear which confounders were adjusted for and why they were included | 11-12, 23-24 |
|  |  | (*b*) Report category boundaries when continuous variables were categorized | – |
|  |  | (*c*) If relevant, consider translating estimates of relative risk into absolute risk for a meaningful time period | – |

Continued on next page

| Other analyses | 17 | Report other analyses done—eg analyses of subgroups and interactions, and sensitivity analyses | Supplementary Data |
| --- | --- | --- | --- |
| **Discussion** |  |  |  |
| Key results | 18 | Summarise key results with reference to study objectives | 13-14 |
| Limitations | 19 | Discuss limitations of the study, taking into account sources of potential bias or imprecision. Discuss both direction and magnitude of any potential bias | 14-15 |
| Interpretation | 20 | Give a cautious overall interpretation of results considering objectives, limitations, multiplicity of analyses, results from similar studies, and other relevant evidence | 13-16 |
| Generalisability | 21 | Discuss the generalisability (external validity) of the study results | 13-15 |
| **Other information** |  |  |  |
| Funding | 22 | Give the source of funding and the role of the funders for the present study and, if applicable, for the original study on which the present article is based | 17 |
